# Targeting Cancer Hallmarks with Epigallocatechin Gallate (EGCG): Mechanistic Basis and Therapeutic Targets

**DOI:** 10.3390/molecules29061373

**Published:** 2024-03-20

**Authors:** Wamidh H. Talib, Dima Awajan, Abdelrahim Alqudah, Razan Alsawwaf, Raha Althunibat, Mahmoud Abu AlRoos, Ala’a Al Safadi, Sharif Abu Asab, Rawan W. Hadi, Lina T. Al Kury

**Affiliations:** 1 Faculty of Allied Medical Sciences, Applied Science Private University, Amman 11931, Jordan; rawanalyasari2001@gmail.com; 2 Department of Clinical Pharmacy and Therapeutics, Applied Science Private University, Amman 11931, Jordan; dimaawajan@gmail.com (D.A.); razan.sawwaf@gmail.com (R.A.); rahaalthunibat@gmail.com (R.A.); mahmoudabualroos@gmail.com (M.A.A.); alaa.safadi9@gmail.com (A.A.S.); shareef.ashour@gmail.com (S.A.A.); 3 Department of Clinical Pharmacy and Pharmacy Practice, Faculty of Pharmaceutical Sciences, The Hashemite University, Zarqa 13133, Jordan; abdelrahim@hu.edu.jo; 4 Department of Health Sciences, College of Natural and Health Sciences, Zayed University, Abu Dhabi 144534, United Arab Emirates

**Keywords:** epigallocatechin gallate (EGCG), cancer hallmarks, anticancer, angiogenesis, metastasis, immune evasion

## Abstract

Epigallocatechin gallate (EGCG) is a catechin, which is a type of flavonoid found in high concentrations in green tea. EGCG has been studied extensively for its potential health benefits, particularly in cancer. EGCG has been found to exhibit anti-proliferative, anti-angiogenic, and pro-apoptotic effects in numerous cancer cell lines and animal models. EGCG has demonstrated the ability to interrupt various signaling pathways associated with cellular proliferation and division in different cancer types. EGCG anticancer activity is mediated by interfering with various cancer hallmarks. This article summarize and highlight the effects of EGCG on cancer hallmarks and focused on the impacts of EGCG on these cancer-related hallmarks. The studies discussed in this review enrich the understanding of EGCG’s potential as a therapeutic tool against cancer, offering a substantial foundation for scientists and medical experts to advance scientific and clinical investigations regarding EGCG’s possibility as a potential anticancer treatment.

## 1. Introduction

Cancer continues to be a leading cause of mortality worldwide, presenting a significant challenge to public health [1]. Over the years, extensive research efforts have been implemented to explore various therapeutic strategies aimed at combating cancer. Among the evolving prospects in cancer treatments, there has been a noteworthy focus on natural compounds originating from plants. This focus is credited to their potential as anticancer agents [2]. One of these compounds is epigallocatechin gallate (EGCG), a polyphenolic catechin found abundantly in green tea [3].

EGCG has attracted scientific interest owing to its remarkable bioactive properties. Including potent antioxidant, anti-inflammatory, and anti-proliferative effects [4]. These characteristics have stimulated investigations into the potential of EGCG in treating and preventing cancer [3]. A growing body of evidence suggests that EGCG may target multiple cancer hallmarks, which are the fundamental biological processes and traits that contribute to cancer development and progression [5].

**Figure 1:**
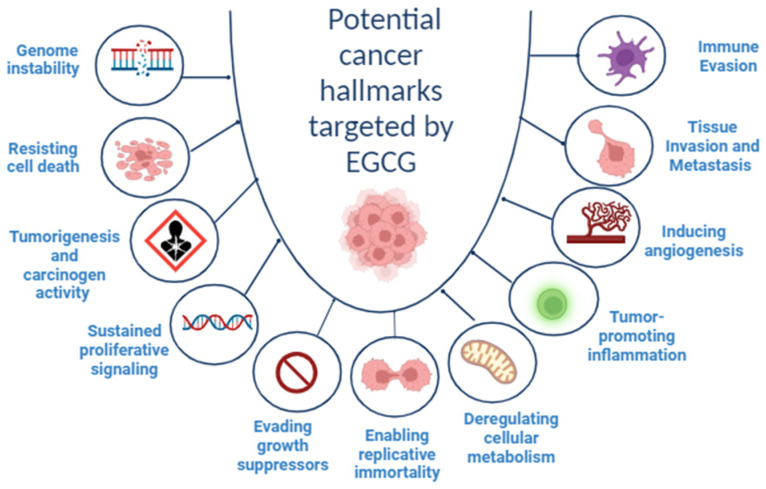
Summary for the potential cancer hallmarks targets for EGCG. Generated by BioRender (https://www.biorender.com/, access date: 1 February 2024).

The cancer hallmarks include sustained proliferative signaling, evasion of growth suppressors, resistance to cell death, replicative immortality, induction of angiogenesis, activation of invasion and metastasis, reprogramming of energy metabolism, and evasion of immune destruction. By targeting these hallmarks, EGCG exhibits a versatile mechanism of action, providing a comprehensive approach to cancer prevention and intervention [6]. Figure 1 shows a summary of cancer hallmarks targeted by EGCG.

This review aims to highlight the potential of EGCG as a promising natural compound for cancer prevention and treatment. Understanding the molecular mechanisms and the impact of EGCG on cancer hallmarks will facilitate the development of novel therapeutic strategies and personalized approaches to improve cancer management.

### 1.1. Pharmacokinetic Properties of EGCG

Unfortunately, most of the studies which reported the pharmacokinetic properties of catechins including EGCG were conducted on animal models rather than humans. In a study conducted to measure the absorption, distribution, and elimination of EGCG in rats, 10 mg/kg of EGCG were intravenously injected. Results showed β-elimination half-live of 135 min, clearance of 72.5 mL/min/kg, and a distribution volume of 22.5 dL/kg [7].

In another study eight subjects, EGCG were given 2 mg/kg pure EGCG or 20 mg decaffeinated green tea. Maximum absorption occurs subsequently to 1.3–1.6 h after ingestion. Moreover, EGCG maximum plasma concentration was 77.9 ± 22.2 at that time [8], with elimination half-live equals to 3.4 ± 0.3.

EGCG absorption takes place in the small intestine, especially in the jejunum and ileum. Therefore, EGCG is transported by paracellular diffusion through the intestinal wall [9]. After the absorption, only a small amount (5%) of the total catechins reaches the plasma. This small amount could be explained by the rapid metabolism and excretion, digestive instability, and poor absorption of the catechins [10]. Following this, up to 90% of EGCG can be found freely unconjugated in the plasma [11].

After EGCG absorption, EGCG is metabolized using methylation by the catechol-O-methyltransferase generating primarily a metabolite called di-methoxyl-EGCG (di-OMe-EGCG) [12]. Sulfo- and/or glucuronic conjugation were also detected during EGCG metabolism. [13]. Moreover, it was proved that intestinal microbiota plays a significant role in EGCG metabolism [14,15].

**Figure 2:**
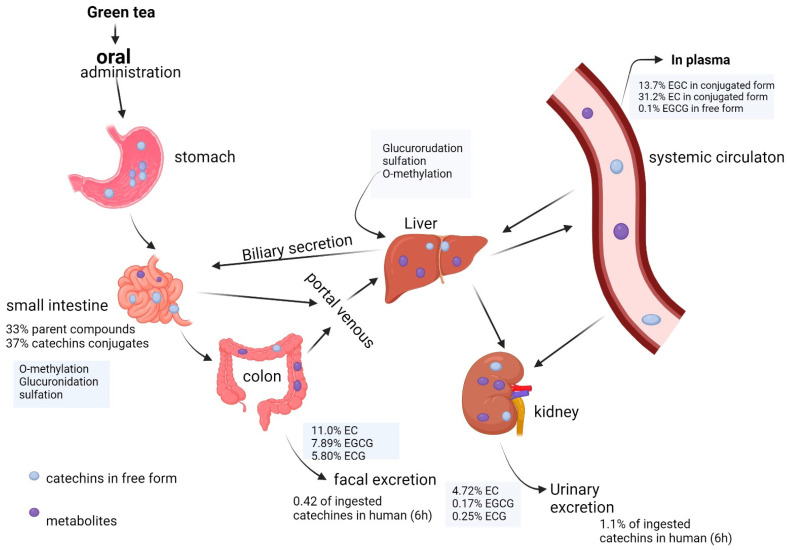
Green tea metabolism in human body [17].

EGCG was found to be excreted in the bile, while other types of catechins such as (−)-epigallocatechin (EGC) and (−)-epicatechin (EC) were excreted in both bile and urine [7]. Figure 2 shows green tea metabolism and distribution in the human body.

It can be concluded that many barriers are challenging EGCG disposition in human bodies. Low blood–brain permeability, poor intestinal stability, and low intestinal absorption are suggested mechanisms to explain the low bioavailability of EGCG [16].

### 1.2. EGCG Bioavailability Obstacles

Oral absorption of EGCG was noticed to be very low, when EGCG was given as oral administration in rats, its absolute bioavailability was 0.1% [18]. Some in vitro studies stated that the active concentration of EGCG was around 1 to 100 μmol/L [18]. Unfortunately, the maximum EGCG concentrations in human plasma were reported to be in lower values (0.16% of ingested catechins) [18]. This can be explained as EGCG is known for its minimal stability and low bioavailability [19].

One of the main reasons behind the low bioavailability of EGCG is intestinal metabolism. Intestinal metabolism affects the bioavailability of flavonoids. It was shown that EGCG was easily metabolized by *Raoultella planticola*, *Enterobacter aerogenes* (*Klebsiella planticola*), *Bifidobacterium longum* subsp., and *K. pneumoniae* subsp. [20]. These types of bacteria are found frequently in gut microbiota [20]. Using the pig cecum model, it was proved that the ester bonds in the gallate derivatives were not durable versus microbial degradation [21]. By using the rat model, after the oral administration of EGCG (25 mg, 54.5 μmol), the fecal metabolites resulted in nine metabolites detected in feces; nevertheless, no EGCG was detected [20]. In addition, there are studies reporting that EGCG may even be degraded and countered by metabolism even before reaching the colon, especially in the small intestine [14]. Therefore, there is an urgent and continuous need for improving EGCG bioavailability.

One of the practices that is utilized to increase EGCG bioavailability is using other natural products such as curcumin and piperine. Curcumin was reported to inhibit P-glycoprotein pump, thus inhibiting EGCG efflux [22], while with piperine, coadministration of EGCG and piperine as 163.8 micromole/kg and 70.2 micromole/kg to male CF-1 mice enhanced the area under the curve and maximum plasma concentration by 1.3-fold related to mice treated with EGCG alone [23]. Furthermore, nanotechnology has improved EGCG bioavailability, EGCG has been prepared using different nano technologies such as nanoemulsion, nanoparticle, and nanoliposome [16]. All these technologies have been shown to increase EGCG bioavailability by different mechanisms. In nanoemulsion, a higher area under the plasma concentration–time curve was detected when rats were treated with tea phenols prepared as nanoemulsions compared to the aqueous form [24]. According to nanoparticles technology, it was proved that when EGCG was encapsulated with chitosan-tripolyphosphate nanoparticles, it resulted in enhancing EGCG oral delivery by increasing the contact of EGCG to the jejunum ended with an upsurge of EGCG plasma concentrations [25]. In nanoliposome technology, it was shown that different concentrations of EGCG altered tumor cell growth in vitro [26]. Moreover, another in vitro study reported that bilosome delivery systems had improved EGCG bioavailability by 1.98 times by enhancing gastrointestinal stability [27]. Also, using microspheres had improved the metabolic stability of EGCG [28].

### 1.3. EGCG Toxicity

Although EGCG is a natural product, it does not mean that it is safe. EGCG can be considered as a safe natural treatment until certain doses are reached. Most of the studies which reported EGCG toxicity were conducted on either mice or rats.

In mice, it was reported that frequent EGCG IP injection (55 or 75 mg/kg/day) for 5 days causes hepatotoxicity and inhibition of hepatic main antioxidant enzymes. And the maximum tolerable dose was 45 mg/kg/day, IP, for 7 days [29]. Moreover, when EGCG was given to diabetic mice at 100 mg/kg/day, intraperitoneally (IP) it caused renal damage [30]. Furthermore, it was noticed that the oral administration of EGCG at 0.5 and 1 g/ kg caused death and adverse effects such as fur loss, abdominal distention, and chattering [31]. Also, specifically in CF-1 mice, it was proved that the single oral administration of EGCG (1500 mg/kg) provoked rigorous hepatotoxicity and death [32]. Additionally, the IP administration of EGCG at 100 mg/kg increased alanine transaminase in mice, while 150 and 200 mg/kg doses triggered death at less than 24 h [33].

Using rat models, when EGCG was used at doses above 10 μmol/L, it resulted in hepatic cellular injury and a decline in hepatocyte function [34]. Another research showed that the oral administration of 2000 mg of EGCG/kg was fatal to rats while 200 mg EGCG/kg was not [35].

**Figure 3:**
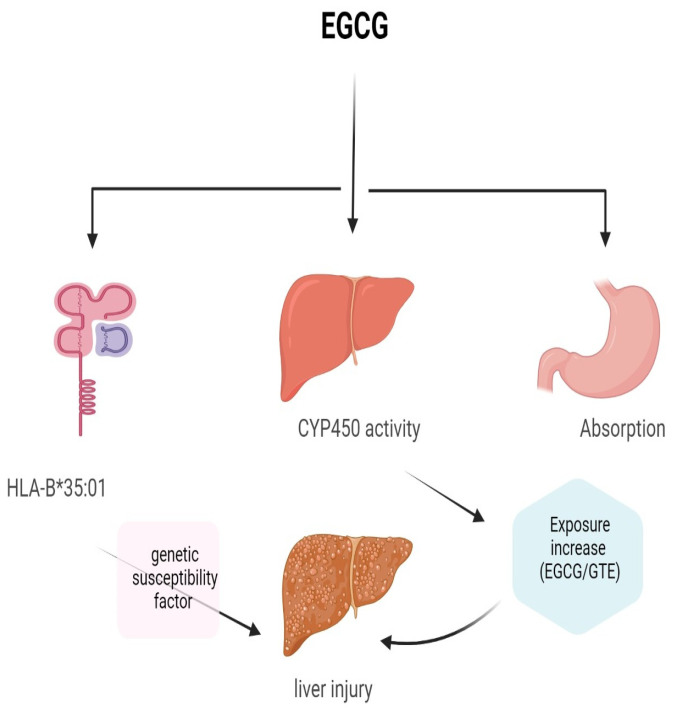
Hepatotoxicity of EGCG [37].

The European food safety authority panel conducted a comprehensive study to the evaluate the possible association between the consumption of EGCG and hepatotoxicity. The study was based on published scientific articles and resulted in the following conclusions: the average daily intake of EGCG from green tea infusion is 90 to 300 mg/day. The levels of EGCG may reach 866 mg/day in high level consumers of green tea herbal infusion and 5–1000 mg /day from food supplements. In humans (NCT00917735 on 8 June 2009), some studies reported that the daily consumption of 0.8 g for 4 months or more may increase the risk of hepatocellular injury [36]. Additionally, starvation is considered as one of the risk factors for green tea hepatotoxicity. Studies showed that dieters carrying the HLA-B*35:01 susceptibility gene have an increased risk of liver injury due to EGCG overconsumption (Figure 3).

## 2. Cancer Hallmarks as Therapeutic Targets

The concept of cancer hallmarks is a dynamic process that progresses continuously with the development in understanding of cancer biology. Recent studies showed that phenotypic plasticity and altered differentiation in cancer cells are also considered among cancer hallmarks [38]. Additionally, specified functional regression, epigenetic factors, microbiome, and neuronal signaling were also proposed as cancer hallmarks [39]. The ongoing advancement of targeted therapies holds promise for improving treatment outcomes and minimizing the side effects associated with traditional non-specific cancer treatments [40].

**Figure 4:**
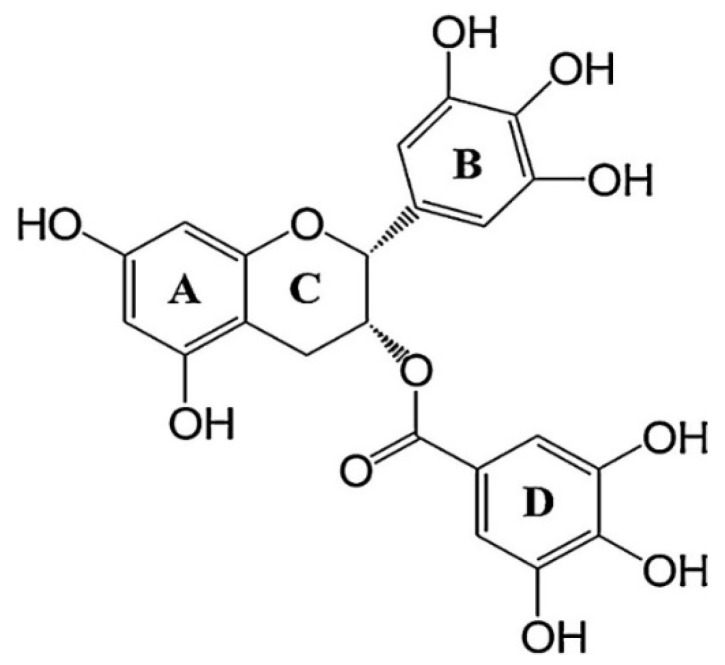
Structure of epigallocatechin-3-gallate. [(2R,3R)-5,7-dihydroxy-2-(3,4,5-trihydroxyphenyl) chroman-3-yl]3,4,5-trihydroxy-benzoate.

EGCG stands as a natural compound that shows promise as a multifaceted therapeutic agent targeting various cancer hallmarks, and ongoing research continues to explore its potential in cancer treatment [6]. The function of EGCG in targeting cancer hallmarks is attributed to its structure (Figure 4). It consists of three aromatic rings (A, B, and D) that are connected by a pyran ring [41]. The transfer of hydrogen atoms or single-electron transfer involving the hydroxyl groups of the B and/or D rings is what gives EGCG its antioxidant properties [42]. In addition, decreasing the number of -OH groups in B and D rings is responsible for the inhibition of proteasome activity in vitro [43]. The study also showed that B-ring/D-ring peracetate-protected EGCG analogs were the most active in proteasome inhibition and apoptosis induction [41]. EGCG promotes anticancer effects by regulating multiple processes such as inhibition of carcinogen activity, tumorigenesis, proliferation, and angiogenesis, and induction of cell death [3].

## 3. EGCG and Cancer Hallmarks

### 3.1. Role of EGCG in Genomic Instability

The cell cycle is a complex sequence of events where replication and division take place. Many regulatory proteins are involved in proper cellular reproduction, including cyclin proteins, cyclin-dependent kinases, oncogenes, tumor-suppressor genes, and mitotic checkpoint proteins [44]. Mutations of any of these regulatory mechanisms can cause the reproduction of cells with genetic mutations or abnormal numbers of chromosomes, resulting in genomic instability [45].

Genomic instability plays a crucial role in the initiation and development of cancer [46]. It refers to an increased propensity for DNA damage, mutations, and chromosomal aberrations within cells [47]. Genomic instability can arise from multiple factors, including errors during DNA replication, exposure to genotoxic agents, defects in DNA repair mechanisms, and alterations in cell cycle checkpoints [45].

In previous studies, five priority targets against genomic instability are identified: (1) prevention of DNA damage; (2) enhancement of DNA repair; (3) targeting deficient DNA repair; (4) impairing centrosome clustering; and (5) inhibition of telomerase activity [48].

In a study conducted on colon adenocarcinoma cells COLO205, it was reported that EGCG in different concentrations (5–40 μg/mL), had variable effects on chromosome instability, initiation of apoptosis, and inhibition of cell division [49]. The study showed that EGCG has selective activity against cancer cells compared with normal cells. It reduced chromosomal instability and inhibited apoptosis in human normal colon epithelial cells. On the other hand, the same concentrations of EGCG induced chromosome instability and apoptosis in colon adenocarcinoma cells [48]. Also, telomeric modulation was suggested as a target for EGCG. It was noticed that EGCG had shortened the telomere and lowered telomerase activity in Caco-2 cells. On the other hand, relatively longer telomeres along with increased methylation were observed in fibroblasts after treatment with EGCG. Also, antioxidant activity was reported at 20 and 200 µM [50]. Furthermore, it was detected that EGCG can bargain some protection against UV-induced DNA damage in cell cultures and human peripheral blood samples [51].

### 3.2. Induction of Apoptosis

#### 3.2.1. Caspase-Dependent Apoptosis

EGCG has shown promising effects in inducing apoptosis in cancer cells in laboratory studies and animal models [3]. It has been found to activate multiple pathways involved in apoptosis, including the activation of caspases and enzymes responsible for initiating and executing the apoptotic process [52]. EGCG increases the expression of tumor suppressor genes, like p53 [53]. EGCG also suppresses receptors or signaling proteins that are important for proliferation pathways in cancer cells, including the epidermal growth factor receptor (EGFR) [54], human epidermal growth factor receptor-2 (HER2) [55], insulin-like growth factor receptor (IGF) [56], and mitogen-activated protein kinase (MAPK) [57].

In MCF-7 breast cancer cells, EGCG at 30 µmol/l restrained the proliferation of MCF-7 cells and encouraged apoptosis in these cells, the underlying mechanism may be related to the P53/Bcl-2 signaling pathway [58]. Moreover, in gastric cancer, using SGC7901 cells, EGCG increased apoptosis rates after treatment with 80 μg/mL in hypoxic conditions [59]. Also, in nasopharyngeal carcinoma, EGCG induces apoptosis through downregulation of Sirtuin 1 (SIRT1) when used at 40 μM [60]. In addition, it was shown that 40 µg/ML of EGCG provoked apoptosis in pancreatic cancer via the expression of Phosphatase and tensin homolog deleted on chromosome 10 (PTEN) [61].

Additionally, a study revealed that EGCG inhibits phosphofructokinase activity, increasing the proapoptotic BH3-only protein expression and decreasing the expression of Bcl-2, which eventually leads to apoptosis in hepatocellular carcinoma cells [62].

Moreover, EGCG (0.5 mM) along with epicatechin (3 mM) can induce an apoptotic effect by the inhibition of the DNL (de novo lipogenesis) pathway, which resulted in the prominent activity of carnitine palmitoyl transferase-1 (CPT-1) mediating apoptosis in HepG2 cells [63].

#### 3.2.2. Caspase-Independent Apoptosis

Caspases play a major role in both intrinsic and extrinsic routes of apoptosis. However, caspase-independent mechanisms also require the apoptosis-induction factor (AIF) and endonuclease G (EndoG). Apoptosis is increased once DNA is cleaved by both AIF and EndoG in the nucleus. In an in vitro study, Lee et al. [64] demonstrated that EGCG causes laryngeal epidermoid carcinoma cells to undergo caspase-independent apoptosis.

### 3.3. Tumorigenesis and Carcinogen Activity Suppression

The phrase tumorigeneses refers to the initial formation of a tumor in the body. Carcinogenesis may be developed by the action of biological, physical, or chemical agents that produce a non-lethal, permanent DNA error on the cell. Even though the mechanisms of EGCG on anti-carcinogenesis and anti-tumorigenesis are not clear, the anticancer impact of EGCG has been reported in multiple cancers [65].

In a study conducted on mice, it was observed that EGCG defends from cisplatin-induced DNA damage and/or inhibits tumor growth directly in lung cancer [66]. Furthermore, EGCG was found to prevent obesity-related liver tumorigenesis in obese diabetic mice. Its effect was mediated by inhibiting the insulin-like growth factor (IGF)/IGF-1 receptor (IGF-1R) axis [67].

### 3.4. Role of EGCG in Sustained Proliferative Signaling

The cellular and molecular mechanisms behind the numerous health benefits of EGCG are still not fully understood, most likely because EGCG affects numerous cellular pathways and thus affects numerous processes at once [68]. The primary signaling pathways that are known to be affected by EGCG treatment in various cancer models are briefly outlined in the following subsections:

#### 3.4.1. ERK and PI3K-Akt Pathways

The extracellular-signal-regulated kinase (ERK) pathway is one of the most investigated pathways modulated by EGCG. ERK is considered a key signaling cassette of the mitogen-activated protein kinase (MAPK). In addition, ERK involves a kinase cascade (RAS-Raf-MEK-ERK) and is activated by a membrane receptor, such as an active receptor tyrosine kinase (RTK), to regulate a wide range of cellular functions. Also, ERK controls nuclear and cytosolic transcription factors, enhancing cell growth, differentiation, and survival [69].

Several investigations in cell cultures and animal models showed that the MAPK pathway was inhibited and suppressed in response to EGCG administration [70], primarily through the reduction in ERK1/2 phosphorylation and the inhibition of RAS and Raf-1 activity and expression [71].

It is known that EGCG interacts with RTK receptors, which control cancer signaling [52]. One of the most important receptors which belong to the RTK family is the epidermal growth factor receptor (EGFR) [72]. Activation of the EGFR is necessary for tumor survival and growth, especially in breast cancer and head and neck squamous cell carcinoma [73,74]. It was detected that EGCG inhibits EGFR activation in carcinoma cells [75]. As a result, the mechanism of the EGCG anticancer effect is based on the suppression of the EGFR signaling pathway, which involves a reduction in Akt and ERK1/2 activation [76].

Examining the phosphatase and tensin homolog deleted on chromosome 10 (PTEN), a regulator of the PI3K/Akt/mTOR signaling cascade, has also been used to demonstrate the anti-tumor effects of EGCG treatment in several malignancies. PTEN blocks the phosphorylation of Akt by PDK1 and the subsequent activation of mTOR. The effects of EGCG on pancreatic and ovarian cancer cells include the reduction in cell growth and the encouragement of apoptotic death through an increase in PTEN expression levels and a concurrent decrease in Akt and mTOR activation by phosphorylation [61,76].

#### 3.4.2. 67-LR Pathway

**Figure 5:**
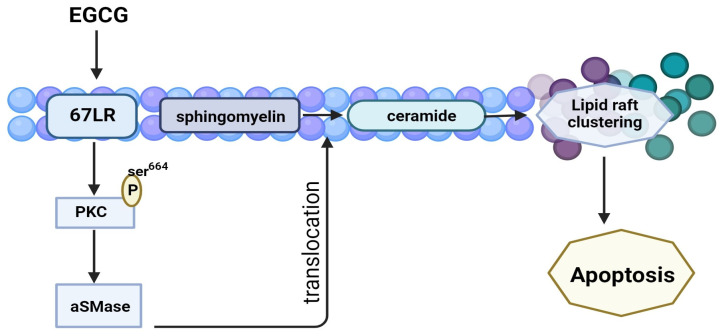
Binding of EGCG induce lipid raft-mediated apoptosis [80].

The 67 kDa high affinity laminin receptor (67LR) is a cell surface receptor for laminin which is the main component of the basement membrane. Binding of this receptor with laminin is essential for cell adhesion, migration, proliferation, and survival. This receptor is overexpressed in many cancer cells, is another detected target of EGCG. Many 67-LR receptors are found in lipid rafts [77]. The clustering of lipid rafts in vast platforms loaded with cholesterol has been linked to abnormal spatial regulation of RTKs, contributing to the emergence and spread of cancer [78]. It is suggested that EGCG may influence lipid rafts-mediated apoptosis via 67-LR in particular cancer cell types such as multiple myeloma [79] (Figure 5).

Endothelial nitric oxide synthase (eNOS), an enzyme that catalyzes the generation of nitric oxide (NO) from L-arginine, may be phosphorylated and activated by the binding of EGCG to 67-LR. The release of NO cause soluble guanylate cyclase (sGC) activation and production of cGMP, which in turn activates PKC. The activated PKC phosphorylate and activates acid sphingomyelinase (SMase), which catalyzes the hydrolysis of sphingomyelin to ceramide (and phosphorylcholine), inducing lipid raft-mediated apoptosis (Figure 3) [52,79]. On the other hand, over production of NO is associated with toxic effects on endothelium. During immune defense mechanisms against infection, NO is produced together with superoxide. The reaction between these two products causes the generation of reactive nitrogen species (RNS). Elevated levels of NO and RNS induce modification of proteins function, alteration in membrane permeability and fluidity, and DNA mutations.

#### 3.4.3. NF-κB Pathway

One of the most studied targets in cancer research is nuclear factor kappa (NF-κB). It is a transcription factor that controls the expression of a wide range of proinflammatory mediators [81]. Nuclear factor-κB is involved in many cellular mechanisms, such as death, growth, inflammation, and immune response [82,83]. NF-κB acts as a regulator of gene transcription in response to oxidative stress [84]. Numerous investigations have proven that EGCG has an inhibitory effect on NF-κB [85,86,87]. In lung cancer, it was reported that a combination of EGCG and the NF-κB inhibitor BAY11-7082 had a synergistic effect in reducing lung cancer cell proliferation by suppressing NF-κB signaling, both in vitro and in vivo, also EGCG alone inhibits lung cancer cell proliferation by suppressing NF-κB signaling [85]. Furthermore, in ovarian cancer, EGCG was noticed to improve cisplatin resistance by altering the Myb-induced NF-κB-STAT3 signaling pathway [88]. Also, in prostate cancer, it was proven that consumption of green tea extract may lower the levels of NF-κB and p53 in rats [89].

In addition, EGCG was utilized at various concentrations to control NF-κB activity to have a protective impact against the negative cascade that was triggered by UV radiation in normal human epidermal keratinocytes [90]. Additionally, it was reported that EGCG reduced cisplatin toxicity on kidney function as it decreased oxidative stress and NF-κB in mice [91].

### 3.5. Role of EGCG in Evasion of Anti-Growth Signaling

Carcinogenesis, the process of cancer development, involves intricate cellular changes and the acquisition of mechanisms to overcome growth suppression [92]. EGCG, a potent polyphenol found in green tea, has shown the potential to interfere with this anti-growth signaling pathway [93]. The role of EGCG in the evasion of anti-growth signaling pathways involves targeting multiple mechanisms. These mechanisms involve genetic alterations, such as chromosomal deletion, mutation, and inactivation of key regulators [94]. Additionally, epigenetic modifications, such as DNA methylation and histone modifications, can contribute to the evasion of tumor suppressors [95]. The loss or dysfunction of tumor suppressor genes, such as CDKN2A, TP53, and PTEN, is frequently observed in solid tumors, leading to unopposed activation of signaling pathways that promote tumor growth [96]. There are several pathways included in the growing process of cancer cells, such AT-rich interactive domain 1A (ARID1A), Hippo, growth differentiation factor 15 (GDF15), insulin-like growth factor (IGF), p53, phosphatase and tensin homolog (PTEN), retinoblastoma protein (Rb), Notch, and Krüppel-like factor 5 (KLF5) pathways [97]. Among these pathways, EGCG showed a proven effect on the Rb pathway. The Rb gene, recognized as one of the earliest tumor suppressor genes, plays a crucial role in cell cycle progression and the integration of various intra- and extracellular signals [98]. Loss or inactivation of the Rb gene, often accompanied by alterations in the cyclin-dependent kinases (CDKs) and E2F family of transcription factors, contributes to the evasion of anti-growth signaling in cancer cells [99]. It was stated that green tea-derived galloyl polyphenol and EGCG were displayed to reduce the phosphorylation of Rb, and as a result, cells were arrested in G1 phase [100].

EGCG inhibits prostate cancer by induction of P53-dependent apoptosis [101]. In another study, a synergistic effect was observed between EGCG and luteolin against head and neck and lung cancer cell lines. This combination caused 3–5-fold increase in apoptosis compared with single treatments [100]. Moreover, it was shown that EGCG inhibited gastric cancer in vitro and in vivo through the activation of apoptosis [102].

### 3.6. Role of EGCG in Replicative Immortality

Telomerase activity is considered as one of the mechanisms responsible for genome stability. The levels of these enzymes vary between different cells and even between cancer cells within the same tumor [103].

The catalytic subunit of telomerase, called hTERT, is crucial for its proper function and has been observed to be expressed in approximately 90% of all cancer types [104].

Accumulating evidence demonstrates that EGCG effectively suppresses telomerase activity in diverse cancer cell types [105]. This inhibition leads to a progressive reduction in telomere length, ultimately impeding replicative potential. Studies have elucidated the molecular mechanisms underlying EGCG’s anti-telomerase effects, including disruption of telomerase assembly and inhibition of telomerase enzymatic activity [105]. Furthermore, EGCG-induced telomere shortening has been correlated with cell cycle arrest and induction of cellular senescence in cancer cells [106].

Several studies reported the ability of EGCG to modulate the expression and activity of telomere-associated proteins [107]. EGCG showed activity through inhibition of telomerase activity, alteration of telomeric DNA-binding proteins, and disruption of telomere-related signaling pathways [108,109].

The effects of EGCG on telomerase activity were observed in several cancer models. It was reported that 20 and 200 µM of EGCG caused telomere shortening and decreased telomerase activity in Caco-2 cells [50]. Moreover, in glioblastoma, EGCG suppressed the growth of U251 cells through telomerase inhibition [106]. Additionally, down regulation of telomerase expression was reported in breast cancer cells after treatment with EGCG [110].

### 3.7. Role of EGCG in Tumor Dysregulated Metabolism

**Figure 6:**
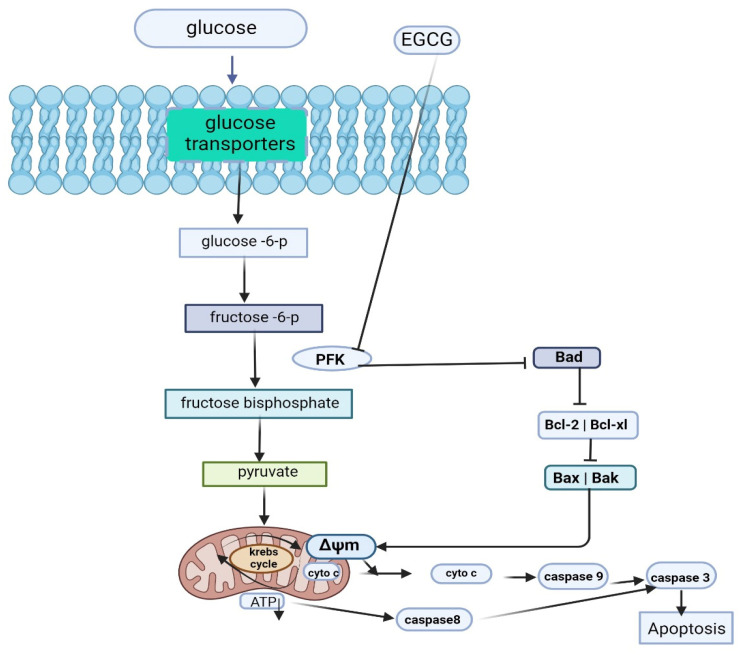
EGCG induce apoptosis in cancer cells through targeting glucose metabolism [62].

EGCG has emerged as a promising candidate for targeting tumor-dysregulated metabolism (Figure 6). Its ability to modulate various metabolic pathways involved in energy production, lipid metabolism, and redox balance highlights its potential as a therapeutic agent for combating cancer [111].

Tumor cells tend to rely on glycolysis for energy production, even in the presence of oxygen. EGCG has been found to inhibit key enzymes involved in glycolysis, such as hexokinase and pyruvate kinase, thereby disrupting the Warburg effect and inhibiting tumor cell growth [112]. It was noticed that a combination of EGCG and gemcitabine reduced pancreatic cancer growth in the xenograft model by 67%, which was explained by suppressing glycolysis [113]. Plus, in colorectal cancer, EGCG treatment of cancer-associated fibroblasts restrained their tumor-promoting abilities by stopping their glycolytic activity [112].

Also, EGCG has been shown to inhibit fatty acid synthase, an enzyme involved in de novo fatty acid synthesis. By suppressing lipid synthesis, EGCG can interfere with the formation of lipid membranes essential for tumor cell proliferation. Additionally, EGCG can activate AMP-activated protein kinase (AMPK), a metabolic regulator that inhibits lipid biosynthesis and promotes fatty acid oxidation [114]. In the literature, it was informed that EGCG sensitizes radioresistance by reducing fatty acid synthase, and EGCG acts as a radiosensitizer for better treatment of nasopharyngeal carcinoma [115]. Moreover, in triple-negative breast cancer, it was stated that EGCG inhibited fatty acid synthase [116].

Glutamine is a vital nutrient for cancer cells, and its metabolism plays a crucial role in supporting tumor growth [117]. EGCG has been found to inhibit glutamine uptake and the activity of glutaminase, an enzyme involved in glutamine metabolism. By disrupting glutamine utilization, EGCG can impede the growth and survival of tumor cells [118]. In Hepatocellular carcinoma, EGCG inhibited glutamate dehydrogenase 1 and blocked cell growth under low glucose conditions [119].

EGCG exhibits antioxidant properties and can modulate cellular redox balance. It can scavenge reactive oxygen species (ROS) and enhance the activity of antioxidant enzymes, such as superoxide dismutase and catalase [120]. By regulating redox homeostasis, EGCG can mitigate oxidative stress, which is often elevated in cancer cells and contributes to tumor progression [121]. In non-small lung cancer cells, EGCG showed a pro-oxidative effect through copper transporter 1 regulation [122]. Moreover, in lung adenocarcinoma, a minimal dose of EGCG (0.5 μM) enhanced Doxorubicin (10 μM) toxicity and revealed oxidative damage-mediated antineoplastic ability by reorienting the redox signaling in A549 cells [123].

### 3.8. Role of EGCG in Tumor-Promoting Inflammation

Increased inflammatory mediators are associated with a bad prognosis in cancer patients, and inflammation is a key factor in the development and progression of cancer [124]. Tumor necrosis factor, chemokines, inflammasomes, transcription factors, cytokines, infiltrating or circulating immune cells, and ROS are a few of the cancer-related inflammatory factors that contribute to the development of cancer [125]. Tumor necrosis factor (TNF) is a pro-inflammatory cytokine associated with different cancer types. This cytokine works by promoting tumor cell migration and invasion also regulating penetration of tumor-associated macrophages into the tumor microenvironment [126].

EGCG has been proven to have anti-inflammatory properties and to block the production of pro-inflammatory cytokines [127]. It was reported that 10 μM and 50 μM of EGCG treatment exhibited inhibitory activity for NO, COX-2, IL-6, IL-1β, and TNF-α in LPS-induced RAW 264.7 cells [128].

COX-2 overexpression has been observed in a variety of malignancies. However, regulating it is a critical step toward cancer management. In androgen-sensitive and androgen-insensitive human prostate cancer cells, EGCG suppresses cyclooxygenase-2 without altering COX-1 expression at both the mRNA and protein levels. Based on these data, it is tempting to suggest that combining EGCG with chemotherapeutic medications might be a superior method for prostate cancer prevention and therapy [129].

### 3.9. Role of EGCG in Angiogenesis Inhibition

A major angiogenic factor is known as vascular endothelial growth factor (VEGF). It is usually upregulated in cancer, as it contributes to forming new blood vessels, thus enhancing tumor survival [130]. Cancer regularly needs a blood supply for development, and when this supply is cut off, cancer can become dormant [131]. The ability to inhibit the angiogenesis process is seen as a strength in cancer therapy efforts. Angiogenesis plays an important part in the development of many health problems [132].

EGCG plays a dynamic starring role in the suppression of uncontrolled angiogenesis by inhibiting Vascular endothelial growth factor (VEGF) [133]. In one study, it was reported that EGCG strongly inhibited VEGF mRNA transcription in gastric cancer at different concentrations (20 μg/mL, 60 μg/mL, and 100 μg/mL) [59]. In breast cancer, EGCG inhibited the protein expression of VEGF when used at 25, 50, and 100 mg/L in vitro [134]. Also, in colorectal cancer, EGCG when used at 0.01 and 1% inhibited the activation of the VEGF/VEGF receptor axis, which is important in the pathological angiogenesis of tumor cells [135]. In addition, in neck and breast carcinoma, EGCG at 30µg/mL EGCG was reported to lower VEGF production in head and neck and breast carcinoma cells by inhibiting epidermal growth factor receptor (EGFR) related pathways [136]. A prodrug derived from EGCG (Pro-EGCG) was tested to inhibit angiogenesis in endometrial cancer. The results showed an anti-angiogenic effect of this drug through the inhibition of the PI3K/AKT/mTOR/HIF1α pathway [137]. Furthermore, it was reported that EGCG potentiates the antiproliferative activity of sunitinib against different cancer cell lines, by reducing the production of VEGF [138]. Moreover, in gastric cancer, it was noticed that EGCG inhibited VEGF secretion and also inhibited the expression of transcription factor activator protein 2A phosphorylation [139]. Besides hepatocellular carcinoma, EGCG inhibited angiogenesis by inhibiting VEGF-dependent pathways [71]. In addition, much research proved that EGCG decreased VEGF in different breast cancer cells [140].

### 3.10. Role of EGCG in Tissue Invasion and Metastasis

Tissue invasion is the process by which cancer cells grow into adjacent environments [141]. Metastasis refers to the process of cancer cells breaking away from the prime tumor, migrating to a new site, and finding a new, or secondary tumor, in the new environment [142].

EGCG had shown a great effect in targeting cancer invasion and metastasis. In colorectal cancer, EGCG inhibited the cancer invasion by downregulating MMP-2 and MMP-9 expression [143]. In addition, in hypopharyngeal carcinoma, EGCG inhibited hepatocyte growth factor-induced invasion [144]. Also, in pancreatic cancer, EGCG reduced the invasion and metastasis in vitro and in vivo [145]. In addition, in highly invasive lung cancer, EGCG inhibited CL1-5 cells invasion and migration. Also, it improved docetaxel effect in suppressing metastasis of these cells [146]. Moreover, in MCF-7 tamoxifen-resistant cells, EGCG stopped cell growth and invasion through the down-regulation of EGFR [147].

### 3.11. Role of EGCG in Tumor-Associated Immune Evasion

The immune system is present to save the body from infections and foreign antigens, but the cancer cell can use a different mechanism to suppress the immune system attack and increase tumor progression all over the body [148]. There are several pathways in which cancer cells can affect the immune system. The two major ways are affecting regulatory cells such as Myeloid-derived suppressor cells [149] or affecting certain pathways such as programmed cell death protein (PD) and programmed cell death ligand (PD-L) related pathways [150].

#### 3.11.1. Myeloid-Derived Suppressor Cells (MDSCs)

Myeloid-derived suppressor cells (MDSCs) are composed of a large and heterogenous group of immature myeloid cell in various transcriptional and differential states. Pathological condition induce the expansion of these myeloid leukocytes to eliminate threats. MDSCs can be divided into two main groups: polymorphonuclear (PMN-MDSC) and monocytic (M-MDSC). The identification of MDSCs cells in human is based on the presence of myeloid markers and the two main groups of can be identified as Lin^−^HLA-DR^−/lo^CD33^+^ or Lin^−^HLA-DR^−/lo^CD11b^+^CD14^−^CD15^+^CD33^+^ for PMN-MDSCs and CD14^+^HLA-DR^neg/lo^ or Lin^−^HLA-DR^neg/lo^CD11b^+^CD14^+^CD15^−^ for M-MDSCs [151]. They have a critical role in tumor progression by suppressing anti-tumor immunity. Mostly, these cells are in peripheral lymphoid organs, and these cells defeat the T cell in a non-specific way. They are the main tumor-favoring and immune suppressor cells that promote tumor metastasis [152]. In the murine breast cancer model, EGCG amended immunosuppression by meaningfully diminishing the accumulation of MDSCs and enhancing the proportions of CD4+ and CD8+ T cells in spleen and tumor sites in 4T1 breast tumor in vivo [153].

#### 3.11.2. Programmed Cell Death Protein (PD) and Programmed Cell Death Ligand (PD-L)

Tumor cells have a complex network of relationships with multiple surrounding components called the tumor microenvironment (TME), which always enhances tumorigenesis and progression. Programmed cell death protein 1(PD-1) and programmed cell death ligand 1 (PD-L-1) are very important immune-negative regulatory compounds in the tumor microenvironment, and the PD-1/PD-L1 pathway was found to be a key player for tumor immune evasion [154].

PD-1 (CD28 family receptor, CD279) is from immunoglobulin superfamily type I transmembrane glycoprotein [155]. As a co-inhibitory receptor, PD-1 is expressed on activated T cells and has two ligands: PD-L1 (B7-H1 or CD274) and PD-L2 (CD273) [156]. Like PD-1, PD-L1 is also from the immunoglobulin superfamily (type I transmembrane protein) and can be expressed in hematopoietic and non-hematopoietic normal tissue cells [157]. Also, PD-L1 expression can be upregulated in many other cell types in response to inflammatory cytokines and other stimuli [158].

PD-1 and PD-L1 are very important for maintaining immune homeostasis and play a major role in T cell co-stimulation and co-inhibition [159]. Under normal physiological conditions, the PD-1/PD-L1 axis saves a balance between tolerance and autoimmunity [160]. Immune activation (e.g., inflammation) leads to the upregulation of multiple immune borders on the T cell surface to reduce the overactivation of immune cells, preserve immune homeostasis, and prevent autoimmune diseases [161]. In patients with chronic inflammation or cancer, PD-1 is a key player in the induction of T cell failure [162].

Using antibodies targeting the PD-1/PD-L1 pathway is one of the special techniques for targeting many cancers such as melanoma, lung, breast, liver, colorectal, lymphoma, head and neck, cervical, and gastric cancers [163]. In addition to other tumors expressing high levels of activated tumor-specific T [164].

EGCG was tested to inhibit cancer by targeting immune checkpoints. The results showed an inhibition of melanoma cells through targeting JAK-STAT signaling and inhibiting IFN-γ-induced PD-L1 and PD-L2 expression [165]. Moreover, EGCG inhibited PD-L1 expression in non-small-cell lung cancer cells [166]. PD-L1 inhibition through EGCG treatment was also reported as a method enhances anti-tumor immunity [167].

In Table 1, there is a summary for all studies that reported the effect of EGCG in different cancer hallmarks and the outcomes for these studies.

## 4. Conclusions

Cancer continues to be a leading cause of mortality and morbidity worldwide despite significant recent advancements. Anticancer medications are successful in treating cancer, but they can have negative side effects, such as alterations in physiological and biochemical processes, exhaustion, hair loss, infection, nausea, and vomiting. The evaluation of numerous biological compounds and natural products has been demonstrated to have a key role in the prevention and suppression of cancer. The most prevalent catechin in tea, epigallocatechin-3-gallate, and its significance in health care and illness prevention have been documented. Many experimental and clinical research studies have noted that EGCG has anticancer properties. It can be used either alone or in conjunction with other treatments to accomplish its inhibitory effects. EGCG is a key physiological anticancer drug due to its role in triggering multiple anticancer processes in many cancer hallmarks. To consider EGCG as a routine therapeutic option to treat various malignancies, more clinical studies are required.

## Figures and Tables

**Table 1:** Experimental design of EGCG in different cancer hallmarks and the outcomes of these studies.

Cancer Hallmark	Concentration Used	Type of Cells	Experimental Model	Outcomes of the Combination	Reference
Genomic Instability	5–40 μg/mL	Colon adenocarcinoma	in vitro	EGCG exhibits different genetic and cytological effects on colon cancer cells.	[49]
	20 and 200 µM	Colorectal adenocarcinoma	in vitro	Telomeric modulation in cancer vs. primary cells and specific antioxidant activity of EGCG against oxidative damage to lipids in abnormal cells.	[50]
	250 μM	Lung fibroblasts, skin fibroblasts, and epidermal keratinocytes	in vitro	Protection against UV-induced DNA damage in human cell cultures and human peripheral blood samples.	[51]
Inducing Apoptosis	30 µmol/L	Breast cancer	in vitro	EGCG suppressed the proliferation of human MCF-7 breast cancer cells and promoted apoptosis through the P53/Bcl-2 signaling pathway.	[58]
	80 μg/mL	Gastric cancer	in vitro	EGCG induces apoptosis of gastric cancer SGC7901 cells via down-regulating HIF-1α and VEGF under a hypoxic state.	[59]
	40 μM	Nasopharyngeal cancer	in vitro	EGCG could inhibit the growth of nasopharyngeal cancer cells through the inhibition of the SIRT1-p53 signaling pathway.	[60]
	40 µg/mL	Pancreatic cancer	in vitro	EGCG was able to inhibit proliferation and induce apoptosis in pancreatic cancer cells via PTEN, with the loss of PTEN reducing the ability of EGCG to inhibit proliferation and promote apoptosis in pancreatic cancer cells.	[61]
	50 and 100 Μm 10 mg/kg/BW/day)	Hepatocellular carcinoma	in vitro in vivo	EGCG directly suppresses Phosphofructokinase activity and induces apoptosis by promoting a metabolic shift away from glycolysis in aerobic glycolytic hepatocellular carcinoma (HCC) cells.	[62]
	0.5 mM	Hepatocellular carcinoma	in vitro	EGCG and epicatechin induced an inhibitory effect on the enzyme expression and activity of the de novo lipogenesis (DNL) pathway, which leads to the prominent activity of carnitine palmitoyl transferase-1 (CPT-1) mediating apoptosis in HepG2 cells.	[63]
Caspase-independent apoptosis	200 µM	Laryngeal epidermoid carcinoma	in vitro	P53-mediated mitochondrial pathway and the nuclear translocation of AIF and EndoG play a crucial role in EGCG-induced apoptosis of human laryngeal epidermoid carcinoma Hep2 cells, which proceeds through a caspase-independent pathway.	[64]
Tumorigenesis and carcinogen activity suppression	1 mg/mL	Lung cancer	in vivo	EGCG inhibits cisplatin-induced weight loss and lung tumorigenesis in A/J mice	[66]
	0.1%	Liver cancer	in vivo	EGCG prevents obesity-related liver tumorigenesis by inhibiting the IGF/IGF-1R axis.	[67]
Sustained Proliferative Signaling	(0, 5, 10, 20, 40 and 80 µg/mL) 10, 30 or 50 mg/kg	Ovarian cancer	in vitro in vivo	The involvement of PTEN/AKT/mTOR signaling pathway in the anti-ovarian cancer effects of EGCG in vitro and in vivo.	[76]
	40 µg/mL	Pancreatic cancer	in vitro	Inhibit proliferation and induce apoptosis in PC cells via PTEN. EGCG can downregulate the expression levels of p-Akt and p-mTOR to regulate the PI3K/Akt/mTOR pathway via PTEN.	[61]
	5–20 μM 20 mg/kg	Multiple myeloma	in vitro in vivo	EGCG induces lipid-raft clustering and apoptotic cell death by activating protein kinase Cδ and acid sphingomyelinase through a 67 kDa laminin receptor.	[79]
	160 μM 20 mg/kg	Lung cancer	in vitro in vivo	EGCG inhibits cell proliferation and migration and induces apoptosis in A549 and H1299 cells. EGCG inhibits lung cancer cell proliferation by suppressing NF-κB signaling.	[85]
	1, 5, and 20 μM	Ovarian cancer	in vitro	Dietary factors EGCG and sulforaphane altered c-Myb-mediated ovarian cancer progression and chemoresistance.	[88]
	1.34 mL of green tea extract	Prostate cancer	in vivo	Longstanding exercise training linked with the consumption of green tea extract may decrease levels of NF-κB and p53 in rats with prostate cancer.	[89]
Evasion of Anti-Growth Signaling	100 mM of EGC	Adenocarcinoma	in vitro	EGC caused a dose-dependent accumulation of cells in the G1 phase and a decrease in the phosphorylation of the retinoblastoma (Rb) protein, which was also in a cellular thiol-dependent manner.	[100]
	40 and 80 μM	Prostate cancer	in vitro	EGCG activates growth arrest and apoptosis primarily via a p53-dependent pathway that involves the function of both p21 and Bax.	[101]
	30 μM 125 mg/kg	Head and neck and lung cancer	in vitro in vivo	A combination of EGCG and luteolin Significantly inhibits Ki-67 expression and boosts TUNEL-positive cells in xenografted tissues. A combination of EGCG and luteolin induced mitochondria-dependent apoptosis in some cell lines and mitochondria-independent apoptosis in others. More efficient stabilization and ATM-dependent Ser15 phosphorylation of p53 due to DNA damage by the combination. Ablation of p53 using shRNA strongly inhibited apoptosis as evidenced by decreased poly (ADP-ribose) polymerase and caspase-3 cleavage. Mitochondrial translocation of p53.	[168]
	30μM 15, 30, and 45 μM	Gastric cancer	in vitro in vivo	EGCG suppressed gastric cancer cell proliferation and demonstrated that this inhibitory effect is related to canonical Wnt/β-catenin signaling.	[102]
Replicative Immortality	20 and 200 µM	Colorectal adenocarcinoma	in vitro	EGCG induced telomere shortening and decreased telomerase activity in Caco-2 cells.	[50]
	1, 5, and 10 µg/mL	Glioblastoma	in vitro	EGCG has the potential to stop the growth of U251 cells due to telomerase inhibition. The effect mainly was on cancerous cells and not normal ones.	[106]
	80 µM	Breast cancer	in vitro	EGCG significantly diminished 0.8, 0.4, and 0.3 gene expression of hTERT.	[110]
Tumor Dysregulated Metabolism	20–100 μM 10 mg/kg	Pancreatic cancer	in vitro in vivo	EGCG and gemcitabine combination reduced pancreatic cancer cell growth by suppressing glycolysis.	[113]
	25, 50, or 100 µ	Colorectal cancer	in vitro	EGCG treatment of cancer-associated fibroblasts overcomes their tumor-promoting abilities by stopping their glycolytic activity.	[112]
	25, 50, 100, and 150 μg/mL 30 μg/kg	Nasopharyngeal carcinoma	in vitro in vivo	EGCG may alert radio resistance by reducing fatty acid synthase and work as a radiosensitizer for better treatment of nasopharyngeal carcinoma.	[115]
	10–140 µM	Breast cancer	in vitro	Significantly blocked fatty acid synthase activity in triple-negative breast cancer.	[116]
	0, 25, 50 and 100 μM	Hepatocellular carcinoma	in vitro	EGCG suppressed cell growth under low glucose conditions. EGCG inhibited glutamate dehydrogenase 1.	[119]
	30 μM and 40 μM 20 mg/kg	Non-small-cell lung cancer	in vitro in vivo	EGCG mediated ROS to regulate copper transporter 1 expression through the ERK1/2/ nuclear paraspeckle assembly transcript 1 (NEAT1) signaling pathway.	[122]
	0.5 μM	Lung adenocarcinoma	in vitro	Low-dose EGCG enhanced Doxorubicin toxicity and revealed oxidative damage-mediated antineoplastic efficacy by reorienting the redox signaling in A549 cells.	[123]
Tumor—Inflammation	10 μM and 50 μM	Macrophage-like, Abelson leukemia virus-transformed cell line	in vitro	Oolong tea ethanol extract and EGCG decreased the assembly of NO, COX-2, IL-6, IL-1β, and TNF-α in active macrophages.	[128]
	10, 25, 50, or 100 μM	Prostate cancer	in vitro	EGCG selectively inhibits COX-2.	[129]
Angiogenesis inhibition	20, 40, and 60 µM	Endometrial cancer	in vivo	EGCG reduced the secretion of VEGFA from cancerous cells and also reduced tumor-associated macrophage-secreted VEGFA in endometrial cancer.	[137]
	50 μmol/L 50 mg/kg	Lung cancer Breast cancer	in vitro in vivo	EGCG along with sunitinib simultaneously inhibits the VEGFR2/ mTOR/VEGF signaling cascade.	[138]
	20μM 25 mg/kg	Gastric cancer	in vitro in vivo	EGCG inhibited VEGF secretion and expression, and its up-stream signal regulator; it also down-regulated the transcription of factor activator protein 2A (TFAP2A).	[139]
	30 μg/mL	Hepatocellular carcinoma	in vitro	EGCG inhibited tumor growth and angiogenesis by the intervention of MAPK/ERK1/2 and PI3K/AKT/HIF-1α/VEGF pathways.	[71]
	20 μg/mL, 60 μg/mL, and 100 μg/mL	Gastric cancer	in vitro	With the increasing EGCG concentration, the expressions of HIF-1α and VEGF proteins were suppressed in hypoxic conditions.	[59]
	25, 50, 100 mg/L	Breast cancer	in vitro	Protein expression of HIF-1α and VEGF declined in a dose-dependent manner in MCF-7 cells pretreated with increasing concentrations of EGCG.	[134]
	0.01 and 0.1%	Colorectal cancer	in vivo	Restraining the activation of the VEGF/VEGFR axis by suppressing the expression of HIF-1 in the xenograft model.	[135]
	30 μg/mL	Neck and breast	in vitro	Lowering VEGF by inhibiting EGFR-related pathways of signal transduction.	[136]
Tissue Invasion and Metastasis	15, 30, 60 μM	Colorectal cancer	in vitro	ECG and EGCG dimers restrained colorectal cancer cell invasion/metastasis, by downregulating MMP-2 and MMP-9 expression via a NOX1/EGFR-dependent mechanism, and through a direct inhibitory effect on MMPs enzyme activity.	[143]
	1 μM	Hypopharyngeal carcinoma	in vitro	EGCG inhibited HGF, MMP-9, and urokinase-type plasminogen activator activities, and also inhibited Akt and Erk pathway.	[144]
	20, 40, and 60 μM 60 mg/kg	Pancreatic cancer	in vitro in vivo	EGCG slowed circulating endothelial growth factor receptor 2 (VEGF-R2). EGCG reduced ERK activity and enhanced p38 and JNK activities. EGCG prevented pancreatic cancer growth, invasion, metastasis, and angiogenesis.	[145]
	2.5, 5, 10, 20, and 40 μM	Lung cancer	in vitro	EGCG suppresses the invasion ability of CL1-5 cells. EGCG induced G2/M arrest at higher doses (30, 40, and 50 μM).	[146]
	50 μg/mL	Tamoxifen-resistant breast cancer	in vitro	EGCG prevented MCF-7 tamoxifen-resistant cells growth and in vitro invasion via down-regulation of EGFR and other molecules associated with aggressive biological behavior.	[147]
Evading immune system	50–350 μg/mL 50 μg/mL, 500 μg/mL, 1000 μg/mL, and 2000 μg/mL	Breast cancer	in vitro in vivo	EGCG reduced the production of related cytokines in MDSCs.	[153]
	10 µM 50 mg/kg	Melanoma	in vitro in vivo	EGCG improves anti-tumor immune responses by reducing JAK-STAT signaling in melanoma. EGCG targeted the PD-L1/PD-L2-PD-1 axis.	[165]
	10 and 50 µM 0.85 g/L of catechins (14% EGCG)	Lung cancer	in vitro	EGCG partially restores T cell activity by inhibiting PD-L1/PD-1 signaling, resulting in the inhibition of lung cancer growth.	[166]

## Data Availability

Data are available within the article.

## References

[1] Fidler M.M., Bray F., Soerjomataram I. (2017). The global cancer burden and human development: A review. Scand. J. Public Health.

[2] Talib W.H., Daoud S., Mahmod A.I., Hamed R.A., Awajan D., Abuarab S.F., Odeh L.H., Khater S., Al Kury L.T. (2022). Plants as a source of anticancer agents: From bench to bedside. Molecules.

[3] Almatroodi S.A., Almatroudi A., Khan A.A., Alhumaydhi F.A., Alsahli M.A., Rahmani A.H. (2020). Potential therapeutic targets of epigallocatechin gallate (EGCG), the most abundant catechin in green tea, and its role in the therapy of various types of cancer. Molecules.

[4] Chen C.-Y., Kao C.-L., Liu C.-M. (2018). The Cancer Prevention, Anti-inflammatory and anti-oxidation of bioactive phytochemicals targeting the TLR4 signaling pathway. Int. J. Mol. Sci..

[5] Lin S.-R., Fu Y.-S., Tsai M.-J., Cheng H., Weng C.-F. (2017). Natural compounds from herbs that can potentially execute as autophagy inducers for cancer therapy. Int. J. Mol. Sci..

[6] Ponte L.G.S., Pavan I.C.B., Mancini M.C.S., Da Silva L.G.S., Morelli A.P., Severino M.B., Bezerra R.M.N., Simabuco F.M. (2021). The hallmarks of flavonoids in cancer. Molecules.

[7] Chen L., Lee M.-J., Li H., Yang C.S. (1997). Absorption, distribution, and elimination of tea polyphenols in rats. Drug Metab. Dispos..

[8] Lee M.-J., Maliakal P., Chen L., Meng X., Bondoc F.Y., Prabhu S., Lambert G., Mohr S., Yang C.S. (2002). Pharmacokinetics of tea catechins after ingestion of green tea and (-)-epigallocatechin-3-gallate by humans: Formation of different metabolites and individual variability. Cancer Epidemiol. Biomark. Prev..

[9] Moore R.J., Jackson K.G., Minihane A.M. (2009). Green tea (*Camellia sinensis*) catechins and vascular function. Br. J. Nutr..

[10] Miyazawa T. (2000). Absorption, metabolism and antioxidative effects of tea catechin in humans. BioFactors.

[11] Manach C., Williamson G., Morand C., Scalbert A., Rémésy C. (2005). Bioavailability and bioefficacy of polyphenols in humans. I. Review of 97 bioavailability studies. Am. J. Clin. Nutr..

[12] Meng X., Sang S., Zhu N., Lu H., Sheng S., Lee M.-J., Ho C.-T., Yang C.S. (2002). Identification and characterization of methylated and ring-fission metabolites of tea catechins formed in humans, mice, and rats. Chem. Res. Toxicol..

[13] Williamson G., Dionisi F., Renouf M.J. (2011). Flavanols from green tea and phenolic acids from coffee: Critical quantitative evaluation of the pharmacokinetic data in humans after consumption of single doses of beverages. Mol. Nutr. Food Res..

[14] Schantz M., Erk T., Richling E. (2010). Metabolism of green tea catechins by the human small intestine. Biotechnol. J..

[15] Legeay S., Rodier M., Fillon L., Faure S., Clere N. (2015). Epigallocatechin gallate: A review of its beneficial properties to prevent metabolic syndrome. Nutrients.

[16] Dai W., Ruan C., Zhang Y., Wang J., Han J., Shao Z., Sun Y., Liang J. (2020). Bioavailability enhancement of EGCG by structural modification and nano-delivery: A review. J. Funct. Foods.

[17] Cai Z.-Y., Li X.-M., Liang J.-P., Xiang L.-P., Wang K.-R., Shi Y.-L., Yang R., Shi M., Ye J.-H., Lu J.-L. (2018). Bioavailability of tea catechins and its improvement. Molecules.

[18] Klinski E., Semov A., Yan X., Alakhov V., Muyzhnek E., Kiselev V.J. (2013). Block copolymer based composition of epigallocatechin-3-gallate with improved oral bioavailability as a way to increase its therapeutic activity. J. Nanomed. Biother. Discov..

[19] Mehmood S., Maqsood M., Mahtab N., Khan M.I., Sahar A., Zaib S., Gul S. (2022). Epigallocatechin gallate: Phytochemistry, bioavailability, utilization challenges, and strategies. J. Food Biochem..

[20] Takagaki A., Nanjo F. (2010). Metabolism of (-)-epigallocatechin gallate by rat intestinal flora. J. Agric. Food Chem..

[21] Slot G.V., Humpf H.-U. (2009). Degradation and metabolism of catechin, epigallocatechin-3-gallate (EGCG), and related compounds by the intestinal microbiota in the pig cecum model. J. Agric. Food Chem..

[22] Alexander A., Qureshi A., Kumari L., Vaishnav P., Sharma M., Saraf S., Saraf S. (2014). Role of herbal bioactives as a potential bioavailability enhancer for active pharmaceutical ingredients. Fitoterapia.

[23] Lambert J.D., Hong J., Kim D.H., Mishin V.M., Yang C.S. (2004). Piperine enhances the bioavailability of the tea polyphenol (−)-epigallocatechin-3-gallate in mice. J. Nutr..

[24] Peng Y., Meng Q., Zhou J., Chen B., Xi J., Long P., Zhang L., Hou R. (2019). Nanoemulsion delivery system of tea polyphenols enhanced the bioavailability of catechins in rats. Food Chem..

[25] Dube A., Nicolazzo J.A., Larson I. (2011). Chitosan nanoparticles enhance the plasma exposure of (−)-epigallocatechin gallate in mice through an enhancement in intestinal stability. Eur. J. Pharm. Sci..

[26] Luo X., Guan R., Chen X., Tao M., Ma J., Zhao J. (2014). Optimization on condition of epigallocatechin-3-gallate (EGCG) nanoliposomes by response surface methodology and cellular uptake studies in Caco-2 cells. Nanoscale Res. Lett..

[27] Wang L., Huang X., Jing H., Ma C., Wang H. (2021). Bilosomes as effective delivery systems to improve the gastrointestinal stability and bioavailability of epigallocatechin gallate (EGCG). Food Res. Int..

[28] Onoue S., Ochi M., Yamada S. (2011). Development of (−)-epigallocatechin-3-gallate (EGCG)-loaded enteric microparticles with intestinal mucoadhesive property. Int. J. Pharm..

[29] Bedrood Z., Rameshrad M., Hosseinzadeh H. (2018). Toxicological effects of *Camellia sinensis* (green tea): A review. Phytother. Res..

[30] Rasheed N.O.A., Ahmed L.A., Abdallah D.M., El-Sayeh B.M. (2017). Nephro-toxic effects of intraperitoneally injected EGCG in diabetic mice: Involvement of oxidative stress, inflammation and apoptosis. Sci. Rep..

[31] Chiou Y.-S., John J.A., Ho C.-T., Pan M.-H., Shahidi F. (2016). Evaluation of chemopreventive effects in colitis-associated colon tumourigenesis and oral toxicity of the lipophilic epigallocatechin gallate-docosahexaenoic acid. J. Funct. Foods.

[32] Lambert J.D., Kennett M.J., Sang S., Reuhl K.R., Ju J., Yang C.S. (2010). Hepatotoxicity of high oral dose (−)-epigallocatechin-3-gallate in mice. Food Chem. Toxicol..

[33] Galati G., Lin A., Sultan A.M., O’Brien P.J. (2006). Cellular and in vivo hepatotoxicity caused by green tea phenolic acids and catechins. Free. Radic. Biol. Med..

[34] Kucera O., Mezera V., Moravcova A., Endlicher R., Lotkova H., Drahota Z., Cervinkova Z. (2015). In vitro toxicity of epigallocatechin gallate in rat liver mitochondria and hepatocytes. Oxidative Med. Cell. Longev..

[35] Isbrucker R., Edwards J., Wolz E., Davidovich A., Bausch J. (2006). Safety studies on epigallocatechin gallate (EGCG) preparations. Part 2: Dermal, acute and short-term toxicity studies. Food Chem. Toxicol..

[36] Younes M., Aggett P., Aguilar F., Crebelli R., Dusemund B., Filipič M., Frutos M.J., Galtier P., Gott D.J.E.J. (2018). Scientific opinion on the safety of green tea catechins. EFSA J..

[37] Rani J., Dhull S.B., Rose P.K., Kidwai M.K. (2024). Drug-induced liver injury and anti-hepatotoxic effect of herbal compounds: A metabolic mechanism perspective. Phytomedicine.

[38] Hanahan D.J. (2022). Hallmarks of cancer: New dimensions. Cancer Discov..

[39] Senga S.S., Grose R.P. (2021). Hallmarks of cancer—The new testament. Open Biol..

[40] Nurgali K., Jagoe R.T., Abalo R. (2018). Editorial: Adverse effects of cancer chemotherapy: Anything new to improve tolerance and reduce sequelae?. Front. Pharmacol..

[41] Zhou S.-D., Huang L., Meng L., Lin Y.-F., Xu X., Dong M.-S. (2020). Soy protein isolate-(-)-epigallocatechin gallate conjugate: Covalent binding sites identification and IgE binding ability evaluation. Food Chem..

[42] Vuolo M.M., Lima V.S., Junior M.R.M. (2019). Phenolic compounds: Structure, classification, and antioxidant power. Bioactive Compounds.

[43] LaLandis-Piwowar K.R., Kuhn D.J., Wan S.B., Chen D., Chan T.H., Dou Q.P. (2005). Evaluation of proteasome-inhibitory and apoptosis-inducing potencies of novel (-)-EGCG analogs and their prodrugs. Int. J. Mol. Med..

[44] Li H., Zimmerman S.E., Weyemi U. (2021). Genomic instability and metabolism in cancer. Int. Rev. Cell Mol. Biol..

[45] Wenzel E.S., Singh A.T.K. (2018). Cell-cycle checkpoints and aneuploidy on the path to cancer. Vivo.

[46] Ui A., Chiba N., Yasui A. (2020). Relationship among DNA double-strand break (DSB), DSB repair, and transcription prevents genome instability and cancer. Cancer Sci..

[47] Vodicka P., Musak L., Vodickova L., Vodenkova S., Catalano C., Kroupa M., Naccarati A., Polivkova Z., Vymetalkova V., Försti A. (2018). Genetic variation of acquired structural chromosomal aberrations. Mutat. Res. Toxicol. Environ. Mutagen..

[48] Ferguson L.R., Chen H., Collins A.R., Connell M., Damia G., Dasgupta S., Malhotra M., Meeker A.K., Amedei A., Amin A. (2015). Genomic instability in human cancer: Molecular insights and opportunities for therapeutic attack and prevention through diet and nutrition. Semin. Cancer Biol..

[49] Ni J., Guo X., Wang H., Zhou T., Wang X. (2018). Differences in the effects of EGCG on chromosomal stability and cell growth between normal and colon cancer cells. Molecules.

[50] Pointner A., Mölzer C., Magnet U., Zappe K., Hippe B., Tosevska A., Tomeva E., Dum E., Gessner D., Lilja S. (2021). The green tea polyphenol EGCG is differentially associated with telomeric regulation in normal human fibroblasts versus cancer cells. Funct. Foods Health Dis..

[51] Morley N., Clifford T., Salter L., Campbell S., Gould D., Curnow A. (2004). The green tea polyphenol (−)-epigallocatechin gallate and green tea can protect human cellular DNA from ultraviolet and visible radiation-induced damage. Photodermatol. Photoimmunol. Photomed..

[52] Ferrari E., Bettuzzi S., Naponelli V. (2022). The potential of epigallocatechin gallate (EGCG) in targeting autophagy for cancer treatment: A narrative review. Int. J. Mol. Sci..

[53] Khan H., Reale M., Ullah H., Sureda A., Tejada S., Wang Y., Zhang Z.-J., Xiao J. (2020). Anti-cancer effects of polyphenols via targeting p53 signaling pathway: Updates and future directions. Biotechnol. Adv..

[54] Minnelli C., Cianfruglia L., Laudadio E., Mobbili G., Galeazzi R., Armeni T. (2021). Effect of epigallocatechin-3-gallate on EGFR signaling and migration in non-small cell lung cancer. Int. J. Mol. Sci..

[55] Lei B., Zhang M., Chen X., Liang B., Xie W., Wang H., Li B. (2022). The effect and mechanism of epigallocatechol gallate combined with trastuzumab on the proliferation of HER2 overexpressing breast cancer cells. J. Pharm. Pract. Serv..

[56] Suresh S.V., Byers D.M.J. (2021). Combined curcumin and EGCG target key markers in hepatocellular and colorectal cancers. J. Restor. Med..

[57] Gan L., Zhong L., Shan Z., Xiao C., Xu T., Song H., Li L., Yang R., Liu B. (2017). Epigallocatechin-3-gallate induces apoptosis in acute promyelocytic leukemia cells via a SHP-1-p38α MAPK-Bax cascade. Oncol. Lett..

[58] Huang C.-Y., Han Z., Li X., Xie H.-H., Zhu S.-S. (2017). Mechanism of EGCG promoting apoptosis of MCF-7 cell line in human breast cancer. Oncol. Lett..

[59] Fu J., Yao J., Wang H., Cui W., Leng J., Ding L., Fan K. (2019). Effects of EGCG on proliferation and apoptosis of gastric cancer SGC7901 cells via down-regulation of HIF-1α and VEGF under a hypoxic state. Eur. Rev. Med. Pharmacol. Sci..

[60] Jiang S., Huang C., Zheng G., Yi W., Wu B., Tang J., Liu X., Huang B., Wu D., Yan T. (2022). EGCG inhibits proliferation and induces apoptosis through downregulation of SIRT1 in nasopharyngeal carcinoma cells. Front. Nutr..

[61] Liu S., Xu Z.L., Sun L., Liu Y., Li C.C., Li H.M., Zhang W., Li C.J., Qin W. (2016). (-)-Epigallocatechin-3-gallate induces apoptosis in human pancreatic cancer cells via PTEN. Mol. Med. Rep..

[62] Li S., Wu L., Feng J., Li J., Liu T., Zhang R., Xu S., Cheng K., Zhou Y., Zhou S. (2016). In vitro and in vivo study of epigallocatechin-3-gallate-induced apoptosis in aerobic glycolytic hepatocellular carcinoma cells involving inhibition of phosphofructokinase activity. Sci. Rep..

[63] Khiewkamrop P., Phunsomboon P., Richert L., Pekthong D., Srisawang P. (2018). Epistructured catechins, EGCG and EC facilitate apoptosis induction through targeting de novo lipogenesis pathway in HepG2 cells. Cancer Cell Int..

[64] Lee J.-H., Jeong Y.-J., Lee S.-W., Kim D., Oh S.-J., Lim H.-S., Oh H.-K., Kim S.-H., Kim W.-J., Jung J.-Y. (2010). EGCG induces apoptosis in human laryngeal epidermoid carcinoma Hep2 cells via mitochondria with the release of apoptosis-inducing factor and endonuclease G. Cancer Lett..

[65] Min K.-j., Kwon T.K. (2014). Anticancer effects and molecular mechanisms of epigallocatechin-3-gallate. Integr. Med. Res..

[66] Mimoto J., Kiura K., Matsuo K., Yoshino T., Takata I., Ueoka H., Kataoka M., Harada M. (2000). (–)-Epigallocatechin gallate can prevent cisplatin-induced lung tumorigenesis in A/J mice. Carcinogenesis.

[67] Shimizu M., Sakai H., Shirakami Y., Yasuda Y., Kubota M., Terakura D., Baba A., Ohno T., Hara Y., Tanaka T. (2011). Preventive effects of (−)-epigallocatechin gallate on diethylnitrosamine-induced liver tumorigenesis in obese and diabetic C57BL/KsJ-db/db mice. Integr. Med. Res..

[68] Kumazoe M., Tachibana H. (2016). Anti-cancer effect of EGCG and its mechanisms. Funct. Foods Health Dis..

[69] Guo Y.J., Pan W.W., Liu S.B., Shen Z.F., Xu Y., Hu L.L. (2020). ERK/MAPK signalling pathway and tumorigenesis. Exp. Ther. Med..

[70] Xu D., Peng S., Guo R., Yao L., Mo H., Li H., Song H., Hu L. (2021). EGCG alleviates oxidative stress and inhibits aflatoxin B_1_ biosynthesis via MAPK signaling pathway. Toxins.

[71] Liao Z.-H., Zhu H.-Q., Chen Y.-Y., Chen R.-L., Fu L.-X., Li L., Zhou H., Zhou J.-L., Liang G.J. (2020). The epigallocatechin gallate derivative Y6 inhibits human hepatocellular carcinoma by inhibiting angiogenesis in MAPK/ERK1/2 and PI3K/AKT/HIF-1α/VEGF dependent pathways. J. Ethnopharmacol..

[72] Sigismund S., Avanzato D., Lanzetti L. (2017). Emerging functions of the EGFR in cancer. Mol. Oncol..

[73] Woods L.T., Jasmer K.J., Forti K.M., Shanbhag V.C., Camden J.M., Erb L., Petris M.J., Weisman G.A. (2020). P2Y2 receptors mediate nucleotide-induced EGFR phosphorylation and stimulate proliferation and tumorigenesis of head and neck squamous cell carcinoma cell lines. Oral Oncol..

[74] Liu X., Adorno-Cruz V., Chang Y.-F., Jia Y., Kawaguchi M., Dashzeveg N.K., Taftaf R., Ramos E.K., Schuster E.J., El-Shennawy L. (2021). EGFR inhibition blocks cancer stem cell clustering and lung metastasis of triple negative breast cancer. Theranostics.

[75] Singh S., Sahadevan R., Roy R., Biswas M., Ghosh P., Kar P., Sonawane A., Sadhukhan S. (2022). Structure-based design and synthesis of a novel long-chain 4′′-alkyl ether derivative of EGCG as potent EGFR inhibitor: In vitro and in silico studies. RSC Adv..

[76] Qin J., Fu M., Wang J., Huang F., Liu H., Huangfu M., Yu D., Liu H., Li X., Guan X. (2020). PTEN/AKT/mTOR signaling mediates anticancer effects of epigallocatechin-3-gallate in ovarian cancer. Oncol. Rep..

[77] Pesapane A., Ragno P., Selleri C., Montuori N. (2017). recent advances in the function of the 67 kda laminin receptor and its targeting for personalized therapy in cancer. Curr. Pharm. Des..

[78] Beloribi-Djefaflia S., Vasseur S., Guillaumond F. (2016). Lipid metabolic reprogramming in cancer cells. Oncogenesis.

[79] Tsukamoto S., Hirotsu K., Kumazoe M., Goto Y., Sugihara K., Suda T., Tsurudome Y., Suzuki T., Yamashita S., Kim Y. (2012). Green tea polyphenol EGCG induces lipid-raft clustering and apoptotic cell death by activating protein kinase Cδ and acid sphingomyelinase through a 67 kDa laminin receptor in multiple myeloma cells. Biochem. J..

[80] Porrini C., Ramarao N., Tran S.-L. (2020). Dr. NO and Mr. Toxic—The versatile role of nitric oxide. Biol. Chem..

[81] D’Acquisto F., May M.J., Ghosh S. (2002). Inhibition of nuclear factor kappa B (NF-B). Mol. Interv..

[82] Alharbi K.S., Fuloria N.K., Fuloria S., Rahman S.B., Al-Malki W.H., Shaikh M.A.J., Thangavelu L., Singh S.K., Allam V.S.R.R., Jha N.K. (2021). Nuclear factor-kappa B and its role in inflammatory lung disease. Chem. Interact..

[83] Verzella D., Pescatore A., Capece D., Vecchiotti D., Ursini M.V., Franzoso G., Alesse E., Zazzeroni F. (2020). Life, death, and autophagy in cancer: NF-κB turns up everywhere. Cell Death Dis..

[84] Lingappan K. (2018). NF-κB in oxidative stress. Curr. Opin. Toxicol..

[85] Zhang L., Xie J., Gan R., Wu Z., Luo H., Chen X., Lu Y., Wu L., Zheng D. (2019). Synergistic inhibition of lung cancer cells by EGCG and NF-κB inhibitor BAY11-7082. J. Cancer.

[86] Luo K.-W., Chen W., Lung W.-Y., Wei X.-Y., Cheng B.-H., Cai Z.-M., Huang W.-R. (2017). EGCG inhibited bladder cancer SW780 cell proliferation and migration both in vitro and in vivo via down-regulation of NF-κB and MMP-9. J. Nutr. Biochem..

[87] La X., Zhang L., Li Z., Li H., Yang Y. (2019). (−)-Epigallocatechin Gallate (EGCG) enhances the sensitivity of colorectal cancer cells to 5-FU by inhibiting GRP78/NF-κB/miR-155-5p/MDR1 pathway. J. Agric. Food Chem..

[88] Tian M., Tian D., Qiao X., Li J., Zhang L. (2019). Modulation of Myb-induced NF-kB-STAT3 signaling and resulting cisplatin resistance in ovarian cancer by dietary factors. J. Cell. Physiol..

[89] Saedmocheshi S., Saghebjoo M., Vahabzadeh Z., Sheikholeslami-Vatani D. (2019). Aerobic Training and Green Tea Extract Protect against N-methyl-N-nitrosourea–induced Prostate Cancer. Med. Sci. Sports Exerc..

[90] Avadhani K.S., Manikkath J., Tiwari M., Chandrasekhar M., Godavarthi A., Vidya S.M., Hariharapura R.C., Kalthur G., Udupa N., Mutalik S. (2017). Skin delivery of epigallocatechin-3-gallate (EGCG) and hyaluronic acid loaded nano-transfersomes for antioxidant and anti-aging effects in UV radiation induced skin damage. Drug Deliv..

[91] Pan H., Chen J., Shen K., Wang X., Wang P., Fu G., Meng H., Wang Y., Jin B. (2015). Mitochondrial modulation by Epigallocatechin 3-Gallate ameliorates cisplatin induced renal injury through decreasing oxidative/nitrative stress, inflammation and NF-KB in mice. PLoS ONE.

[92] Peters J.M., Gonzalez F.J. (2018). The evolution of carcinogenesis. Toxicol. Sci..

[93] Sharifi-Rad M., Pezzani R., Redaelli M., Zorzan M., Imran M., Ahmed Khalil A., Salehi B., Sharopov F., Cho W.C., Sharifi-Rad J. (2020). Preclinical activities of epigallocatechin gallate in signaling pathways in cancer. Molecules.

[94] Boumahdi S., de Sauvage F.J. (2020). The great escape: Tumour cell plasticity in resistance to targeted therapy. Nat. Rev. Drug Discov..

[95] Nirmaladevi R., Ilango S., Paital B., Jayachandran P., Padma P.R. (2020). Epigenetic alterations in cancer. Front. Biosci..

[96] Lin L.L., Choucair K., Patel R. (2019). NF1 in Solid Tumors: The Unknown Soldier of Tumor Suppressor Genes?. Genet. Mol. Med..

[97] Amin A.R., Karpowicz P.A., Carey T.E., Arbiser J., Nahta R., Chen Z.G., Dong J.-T., Kucuk O., Khan G.N., Huang G.S. (2015). Evasion of anti-growth signaling: A key step in tumorigenesis and potential target for treatment and prophylaxis by natural compounds. Semin. Cancer Biol..

[98] Singh L., Kashyap S. (2018). Update on pathology of retinoblastoma. Int. J. Ophthalmol..

[99] Ettl T., Schulz D., Bauer R.J. (2022). The renaissance of cyclin dependent kinase inhibitors. Cancers.

[100] Kennedy D.O., Kojima A., Moffatt J., Yamagiwa H., Yano Y., Hasuma T., Otani S., Matsui-Yuasa I. (2002). Cellular thiol status-dependent inhibition of tumor cell growth via modulation of retinoblastoma protein phosphorylation by (−)-epigallocatechin. Cancer Lett..

[101] Hastak K., Agarwal M.K., Mukhtar H., Agarwal M.L. (2005). Ablation of either p21 or Bax prevents p53-dependent apoptosis induced by green tea polyphenol epigallocatechin-3-gallate. FASEB J..

[102] Yang C., Du W., Yang D. (2016). Inhibition of green tea polyphenol EGCG((−)-epigallocatechin-3-gallate) on the proliferation of gastric cancer cells by suppressing canonical wnt/β-catenin signalling pathway. Int. J. Food Sci. Nutr..

[103] Hegde M.R., Crowley M.R. (2019). Genome and gene structure. Emery and Rimoin’s Principles and Practice of Medical Genetics and Genomics.

[104] Slusher A.L., Kim J.J., Ludlow A.T. (2020). The role of alternative rna splicing in the regulation of htert, telomerase, and telomeres: Implications for cancer therapeutics. Cancers.

[105] Eitsuka T., Nakagawa K., Kato S., Ito J., Otoki Y., Takasu S., Shimizu N., Takahashi T., Miyazawa T. (2018). Modulation of telomerase activity in cancer cells by dietary compounds: A review. Int. J. Mol. Sci..

[106] Udroiu I., Marinaccio J., Sgura A. (2019). Epigallocatechin-3-gallate induces telomere shortening and clastogenic damage in glioblastoma cells. Environ. Mol. Mutagen..

[107] Watanabe L.M., Noronha N.Y., Pinhel M.A., Nonino C.B. (2022). Green Tea and Telomere Length Regulation in Health Conditions. Tea as a Food Ingredient.

[108] Chen X., Tang W., Shi J.B., Liu M.M., Liu X. (2019). Therapeutic strategies for targeting telomerase in cancer. Med. Res. Rev..

[109] Shen Z., Wang Y., Wang G., Gu W., Zhao S., Hu X., Liu W., Cai Y., Ma Z., Gautam R.K. (2023). Research progress of small-molecule drugs in targeting telomerase in human cancer and aging. Chem. Interact..

[110] Moradzadeh M., Hosseini A., Erfanian S., Rezaei H. (2017). Epigallocatechin-3-gallate promotes apoptosis in human breast cancer T47D cells through down-regulation of PI3K/AKT and Telomerase. Pharmacol. Rep..

[111] Tauber A.L., Schweiker S.S., Levonis S.M. (2019). From tea to treatment; epigallocatechin gallate and its potential involvement in minimizing the metabolic changes in cancer. Nutr. Res..

[112] Chen S., Nishi M., Morine Y., Shimada M., Tokunaga T., Kashihara H., Takasu C., Yamada S., Wada Y. (2022). Epigallocatechin-3-gallate hinders metabolic coupling to suppress colorectal cancer malignancy through targeting aerobic glycolysis in cancer-associated fibroblasts. Int. J. Oncol..

[113] Wei R., Hackman R.M., Wang Y., Mackenzie G.G. (2019). Targeting glycolysis with epigallocatechin-3-gallate enhances the efficacy of chemotherapeutics in pancreatic cancer cells and xenografts. Cancers.

[114] Chen R., Lai X., Xiang L., Li Q., Sun L., Lai Z., Li Z., Zhang W., Wen S., Cao J. (2022). Aged green tea reduces high-fat diet-induced fat accumulation and inflammation via activating the AMP-activated protein kinase signaling pathway. Food Nutr. Res..

[115] Chen J., Zhang F., Ren X., Wang Y., Huang W., Zhang J., Cui Y. (2020). Targeting fatty acid synthase sensitizes human nasopharyngeal carcinoma cells to radiation via downregulating frizzled class receptor 10. Cancer Biol. Med..

[116] Crous-Masó J., Palomeras S., Relat J., Camó C., Martínez-Garza Ú., Planas M., Feliu L., Puig T. (2018). (−)-Epigallocatechin 3-gallate synthetic analogues inhibit fatty acid synthase and show anticancer activity in triple negative breast cancer. Molecules.

[117] Choi Y.-K., Park K.-G. (2018). Targeting glutamine metabolism for cancer treatment. Biomol. Ther..

[118] Li C., Feng Y., Wang W., Xu L., Zhang M., Yao Y., Wu X., Zhang Q., Huang W., Wang X. (2023). Targeting Glutaminolysis to Treat Multiple Myeloma: An In Vitro Evaluation of Glutaminase Inhibitors Telaglenastat and Epigallocatechin-3-gallate. Anti-Cancer Agents Med. Chem..

[119] Zhou Y., Yu H., Cheng S., Chen Y., He L., Ren J., He X., Chen J., Zheng L., Li F. (2022). Glutamate dehydrogenase 1 mediated glutaminolysis sustains HCC cells survival under glucose deprivation. J. Cancer.

[120] Sznarkowska A., Kostecka A., Meller K., Bielawski K.P. (2017). Inhibition of cancer antioxidant defense by natural compounds. Oncotarget.

[121] Hayes J.D., Dinkova-Kostova A.T., Tew K.D. (2020). Oxidative stress in cancer. Cancer Cell.

[122] Chen A., Jiang P., Zeb F., Wu X., Xu C., Chen L., Feng Q. (2020). EGCG regulates CTR1 expression through its pro-oxidative property in non-small-cell lung cancer cells. J. Cell. Physiol..

[123] Datta S., Sinha D.J. (2022). Low dose epigallocatechin-3-gallate revives doxorubicin responsiveness by a redox-sensitive pathway in A549 lung adenocarcinoma cells. J. Biochem. Mol. Toxicol..

[124] Singh N., Baby D., Rajguru J.P., Patil P.B., Thakkannavar S.S., Pujari V.B. (2019). Inflammation and cancer. Ann. Afr. Med..

[125] Zhao H., Wu L., Yan G., Chen Y., Zhou M., Wu Y., Li Y. (2021). Inflammation and tumor progression: Signaling pathways and targeted intervention. Signal Transduct. Target. Ther..

[126] Waters J.P., Pober J.S., Bradley J.R. (2013). Tumour necrosis factor and cancer. J. Pathol..

[127] Mokra D., Joskova M., Mokry J. (2020). The Role of EGCG in Green Tea Down Regulating Pro-inflammatory Cytokines Systemically. Int. J. Mol. Sci..

[128] Novilla A., Djamhuri D.S., Nurhayati B., Rihibiha D.D., Afifah E., Widowati W. (2017). Anti-inflammatory properties of oolong tea (*Camellia sinensis*) ethanol extract and epigallocatechin gallate in LPS-induced RAW 264.7 cells. Asian Pac. J. Trop. Biomed..

[129] Hussain T., Gupta S., Adhami V.M., Mukhtar H. (2005). Green tea constituent epigallocatechin-3-gallate selectively inhibits COX-2 without affecting COX-1 expression in human prostate carcinoma cells. Int. J. Cancer.

[130] Yang Y., Cao Y. (2022). The impact of VEGF on cancer metastasis and systemic disease. Semin. Cancer Biol..

[131] Anderson N.M., Simon M.C. (2020). The tumor microenvironment. Curr. Biol..

[132] Salajegheh A. (2016). Angiogenesis in Health, Disease and Malignancy.

[133] Rashidi B., Malekzadeh M., Goodarzi M., Masoudifar A., Mirzaei H. (2017). Green tea and its anti-angiogenesis effects. Biomed. Pharmacother..

[134] Luo H., Xu M., Zhong W., Cui Z., Liu F., Zhou K., Li X. (2014). EGCG decreases the expression of HIF-1α and VEGF and cell growth in MCF-7 breast cancer cells. J. BUON.

[135] Shimizu M., Shirakami Y., Sakai H., Yasuda Y., Kubota M., Adachi S., Tsurumi H., Hara Y., Moriwaki H. (2010). (−)-Epigallocatechin gallate inhibits growth and activation of the VEGF/VEGFR axis in human colorectal cancer cells. Chem. Biol. Interact..

[136] Masuda M., Suzui M., Lim J.T., Deguchi A., Soh J.W., Weinstein I.B. (2002). Epigallocatechin-3-gallate decreases VEGF production in head and neck and breast carcinoma cells by inhibiting EGFR-related pathways of signal transduction. J. Exp. Ther. Oncol..

[137] Wang J., Man G.C.W., Chan T.H., Kwong J., Wang C.C. (2018). A prodrug of green tea polyphenol (–)-epigallocatechin-3-gallate (Pro-EGCG) serves as a novel angiogenesis inhibitor in endometrial cancer. Cancer Lett..

[138] Zhou Y., Tang J., Du Y., Ding J., Liu J.-Y. (2016). The green tea polyphenol EGCG potentiates the antiproliferative activity of sunitinib in human cancer cells. Tumor Biol..

[139] Tang H., Zeng L., Wang J., Zhang X., Ruan Q., Wang J., Cui S., Yang D. (2017). Reversal of 5-fluorouracil resistance by EGCG is mediate by inactivation of TFAP2A/VEGF signaling pathway and down-regulation of MDR-1 and P-gp expression in gastric cancer. Oncotarget.

[140] Xiang L.-P., Wang A., Ye J.-H., Zheng X.-Q., Polito C.A., Lu J.-L., Li Q.-S., Liang Y.-R. (2016). Suppressive effects of tea catechins on breast cancer. Nutrients.

[141] Krakhmal N.V., Zavyalova M., Denisov E., Vtorushin S., Perelmuter V. (2015). Cancer invasion: Patterns and mechanisms. Acta Naturae.

[142] Fares J., Fares M.Y., Khachfe H.H., Salhab H.A., Fares Y. (2020). Molecular principles of metastasis: A hallmark of cancer revisited. Signal Transduct. Target. Ther..

[143] Zhu W., Oteiza P.I. (2023). NADPH oxidase 1: A target in the capacity of dimeric ECG and EGCG procyanidins to inhibit colorectal cancer cell invasion. Redox Biol..

[144] Lim Y.C., Park H.Y., Hwang H.S., Kang S.U., Pyun J.H., Lee M.H., Choi E.C., Kim C.-H. (2008). (−)-Epigallocatechin-3-gallate (EGCG) inhibits HGF-induced invasion and metastasis in hypopharyngeal carcinoma cells. Cancer Lett..

[145] Shankar S., Ganapathy S., Hingorani S.R., Srivastava R.K. (2008). EGCG inhibits growth, invasion, angiogenesis and metastasis of pancreatic cancer. Front. Biosci..

[146] Deng Y.-T., Lin J.-K. (2011). EGCG inhibits the invasion of highly invasive CL1-5 lung cancer cells through suppressing MMP-2 expression via JNK signaling and induces G2/M arrest. J. Agric. Food Chem..

[147] Farabegoli F., Papi A., Orlandi M. (2010). (–)-Epigallocatechin-3-gallate down-regulates EGFR, MMP-2, MMP-9 and EMMPRIN and inhibits the invasion of MCF-7 tamoxifen-resistant cells. Biosci. Rep..

[148] Shore N.D. (2015). Advances in the understanding of cancer immunotherapy. BJU Int..

[149] Vinay D.S., Ryan E.P., Pawelec G., Talib W.H., Stagg J., Elkord E., Lichtor T., Decker W.K., Whelan R.L., Kumara H.S. (2015). Immune evasion in cancer: Mechanistic basis and therapeutic strategies. Semin. Cancer Biol..

[150] Zhang L., Zhang M., Xu J., Li S., Chen Y., Wang W., Yang J., Li S., Gu M. (2020). The role of the programmed cell death protein-1/programmed death-ligand 1 pathway, regulatory T cells and T helper 17 cells in tumor immunity: A narrative review. Ann. Transl. Med..

[151] Gabrilovich D.I. (2017). Myeloid-derived suppressor cells. Cancer Immunol. Res..

[152] Condamine T., Ramachandran I., Youn J.-I., Gabrilovich D.I. (2015). Regulation of tumor metastasis by myeloid-derived suppressor cells. Annu. Rev. Med..

[153] Xu P., Yan F., Zhao Y., Chen X., Sun S., Wang Y., Ying L. (2020). Green tea polyphenol EGCG attenuates MDSCs-mediated immunosuppression through canonical and non-canonical pathways in a 4T1 murine breast cancer model. Nutrients.

[154] Liu K., Sun Q., Liu Q., Li H., Zhang W., Sun C. (2022). Focus on immune checkpoint PD-1/PD-L1 pathway: New advances of polyphenol phytochemicals in tumor immunotherapy. Biomed. Pharmacother..

[155] Kantekure K., Yang Y., Raghunath P., Schaffer A., Woetmann A., Zhang Q., Odum N., Wasik M. (2012). Expression patterns of the immunosuppressive proteins PD-1/CD279 and PD-L1/CD274 at different stages of cutaneous T-cell lymphoma (CTCL)/mycosis fungoides (MF). Am. J. Dermatopathol..

[156] Blank C., Mackensen A. (2007). Contribution of the PD-L1/PD-1 pathway to T-cell exhaustion: An update on implications for chronic infections and tumor evasion. Cancer Immunol. Immunother..

[157] Liang S.C., Latchman Y.E., Buhlmann J.E., Tomczak M.F., Horwitz B.H., Freeman G.J., Sharpe A.H. (2003). Regulation of PD-1, PD-L1, and PD-L2 expression during normal and autoimmune responses. Eur. J. Immunol..

[158] Hartley G., Faulhaber E., Caldwell A., Coy J., Kurihara J., Guth A., Regan D., Dow S. (2017). Immune regulation of canine tumour and macrophage PD-L1 expression. Vet. Comp. Oncol..

[159] Hui E., Cheung J., Zhu J., Su X., Taylor M.J., Wallweber H.A., Sasmal D.K., Huang J., Kim J.M., Mellman I. (2017). T cell costimulatory receptor CD28 is a primary target for PD-1-mediated inhibition. Science.

[160] Liang B., Hu X., Ding Y., Liu M. (2021). Tumor-derived exosomes in the PD-1/PD-L1 axis: Significant regulators as well as promising clinical targets. J. Cell. Physiol..

[161] Dong C. (2021). Cytokine regulation and function in T cells. Annu. Rev. Immunol..

[162] Peña-Asensio J., Calvo H., Torralba M., Miquel J., Sanz-de-Villalobos E., Larrubia J.-R. (2021). Anti-Pd-1/Pd-L1 based combination immunotherapy to boost antigen-specific CD8+ T cell response in hepatocellular carcinoma. Cancers.

[163] Yu X., Fang C., Zhang K., Su C. (2022). Recent advances in nanoparticles-based platforms targeting the PD-1/PD-L1 pathway for cancer treatment. Pharmaceutics.

[164] Johnson D., Ma B.B. (2021). Targeting the PD-1/PD-L1 interaction in nasopharyngeal carcinoma. Oral Oncol..

[165] Menon D.R., Li Y., Yamauchi T., Osborne D.G., Vaddi P.K., Wempe M.F., Zhai Z., Fujita M. (2021). EGCG inhibits tumor growth in melanoma by targeting JAK-STAT signaling and its downstream PD-L1/PD-L2-PD1 axis in tumors and enhancing cytotoxic T-cell responses. Pharmaceuticals.

[166] Rawangkan A., Wongsirisin P., Namiki K., Iida K., Kobayashi Y., Shimizu Y., Fujiki H., Suganuma M. (2018). Green tea catechin is an alternative immune checkpoint inhibitor that inhibits PD-l1 expression and lung tumor growth. Molecules.

[167] Rawangkan A., Iida K., Sakai R., Fujiki H., Suganuma M. (2017). Abstract 2665: Green tea catechin, EGCG, enhances antitumor immunity by down-regulation of PD-L1 expression in non-small human lung cancer cell lines. Cancer Res..

[168] Amin A.R., Wang D., Zhang H., Peng S., Shin H.J.C., Brandes J.C., Tighiouart M., Khuri F.R., Chen Z.G., Shin D.M. (2010). Enhanced anti-tumor activity by the combination of the natural compounds (−)-epigallocatechin-3-gallate and luteolin: Potential role of p53. J. Biol. Chem..

